# Cerebrovascular Reactivity After Sport Concussion: From Acute Injury to 1 Year After Medical Clearance

**DOI:** 10.3389/fneur.2020.00558

**Published:** 2020-07-14

**Authors:** Nathan W. Churchill, Michael G. Hutchison, Simon J. Graham, Tom A. Schweizer

**Affiliations:** ^1^Keenan Research Centre for Biomedical Science of St. Michael's Hospital, Toronto, ON, Canada; ^2^Neuroscience Research Program, St. Michael's Hospital, Toronto, ON, Canada; ^3^Faculty of Kinesiology and Physical Education, University of Toronto, Toronto, ON, Canada; ^4^Department of Medical Biophysics, University of Toronto, Toronto, ON, Canada; ^5^Physical Sciences Platform, Sunnybrook Health Sciences Centre, Sunnybrook Research Institute, Toronto, ON, Canada; ^6^Faculty of Medicine (Neurosurgery) University of Toronto, Toronto, ON, Canada; ^7^The Institute of Biomaterials & Biomedical Engineering (IBBME) at the University of Toronto, Toronto, ON, Canada

**Keywords:** BOLD fMRI, cerebrovascular reactivity, longitudinal study, concussion, brain injury

## Abstract

Neuroimaging has identified significant disturbances in cerebrovascular reactivity (CVR) in the early symptomatic phase of sport-related concussion. However, less is known about how whole-brain alterations in CVR evolve after concussion and whether they remain present beyond medical clearance to return to play (RTP). In the present study, CVR was evaluated using blood-oxygenation-level-dependent functional magnetic resonance imaging (BOLD fMRI) during a respiratory challenge. Imaging data were collected for 110 university-level athletes, including 39 concussed athletes and 71 athletic controls. The concussed athletes were imaged at the acute phase of injury (1–7 days post-injury), the subacute phase (8-14 days post-injury), medical clearance to RTP, 1 month post-RTP, and 1 year post-RTP. Enhanced negative BOLD response to controlled breathing was seen at acute injury, with attenuation of the effect mainly occurring by 1 year post-RTP. Secondary analyses showed that greater symptom severity and prolonged recovery were associated with enhanced BOLD response in the acute phase of injury, but a more attenuated BOLD response in the subacute phase. This study provides novel information characterizing the CVR response after concussion and shows CVR to be a sensitive technique for evaluating long-term brain recovery.

## Introduction

Concussion is a form of mild traumatic brain injury (TBI) that manifests as functional disturbances, usually in the absence of overt radiological findings. At present, the diagnosis and management of sport-related concussion is based on symptom status, along with brief evaluations of cognition and balance. Based on international consensus guidelines (1), the medical clearance to return to play (RTP) is similarly determined by symptom resolution, following the completion of a graded exercise protocol. However, the physiological mechanisms that underlie post-concussion impairments remain incompletely understood (2). Moreover, it is presently unclear which physiological changes have dissipated at the time of RTP and which ones persist beyond medical clearance.

There is growing evidence that disturbances in cerebrovascular function play a key role in concussion-related functional impairments (3). Healthy brain function requires precisely regulated cerebral blood flow (CBF) to ensure the delivery of glucose and oxygen to brain tissues in the presence of time-varying neurometabolic demands and other physiological factors, including fluctuations in arterial blood gases and blood pressure (4). The regulation of CBF is achieved through rapid changes in arteriolar vessel diameter, which in turn alter cerebrovascular resistance. These regulatory mechanisms are highly sensitive to TBI and show dysfunction following both concussion and more severe forms of TBI (5, 6). The dysfunction includes impairments in cerebrovascular reactivity (CVR), which is defined as the ability of cerebral blood vessels to constrict and/or dilate in response to vasoactive stimuli.

Blood-oxygenation-level-dependent functional magnetic resonance imaging (BOLD fMRI) provides a sensitive tool for assessing whole-brain CVR, based on the endogenous signal contrast between paramagnetic deoxyhemoglobin and diamagnetic oxyhemoglobin. Brain maps of CVR have been obtained for both healthy and clinical cohorts, typically by manipulating bloodstream carbon dioxide (CO_2_), a potent vasodilator, and measuring the corresponding BOLD signal changes (7). Externally directed changes in respiration, including paced breathing and breath holding, provide simple but effective methods for modulating CO_2_ levels. More accurate CVR measurements may be attained using blended gas delivery systems (8, 9), but simple breathing manipulations do not require specialized equipment, have minimal setup time, and may be well-tolerated by symptomatic individuals. Previous BOLD fMRI studies have identified effects of concussion on CVR in the initial week following a mild TBI (10–12), and others have reported CVR impairments in participants' scanned months post-injury (13, 14). Although these findings highlight the sensitivity of BOLD fMRI in assessing CVR, there has been limited longitudinal examination of concussion-related brain changes relative to RTP, making it unclear whether CVR recovery lags behind symptom resolution.

In a recent study, we examined the CVR of symptomatic concussed athletes within the first week post-injury, with comparison to control athletes, using BOLD fMRI combined with a respiratory challenge (12). In the previous study, concussed athletes showed enhanced negative BOLD response during the controlled breathing phase of the respiratory task. In addition, concussed athletes with greater symptom severity had a greater amplitude of BOLD response. The present study significantly expands on these findings by imaging concussed athletes longitudinally from the acute phase of injury (1–7 days post-injury) to 1 year post-RTP, along with a large normative cohort of control athletes. The effects of concussion were evaluated longitudinally by modeling changes in CVR response across the imaging sessions, relative to acute injury. The CVR response of concussed athletes was also compared cross-sectionally to matched controls at each imaging session. Secondary analyses in the concussed group evaluated the impact of clinical covariates on CVR recovery, including acute symptom severity and time to RTP.

## Methods

### Study Participants

One hundred ten athletes participated in the imaging study. Thirty-nine concussed athletes were recruited consecutively from university-level sport teams at a single institution (including volleyball, hockey, soccer, football, rugby, basketball, lacrosse, and water polo; see Appendix 1 for athlete numbers by sport) through the academic sport medicine clinic, following concussion diagnosis. Diagnosis was determined by a staff physician after sustaining direct or indirect contact to the head with signs and/or symptoms, as per the Concussion in Sport Group guidelines (1). Imaging was conducted at the acute phase of injury (ACU; 1–7 days post-injury), the subacute phase (SUB; 8–14 days post-injury), medical clearance to RTP (RTP), 1 month post-RTP (1MO), and 1 year post-RTP (1YR). Within the longitudinal design, some of the concussed athletes had missed imaging sessions. The number of participants retained at each session was as follows: ACU (23/39), SUB (24/39), RTP (27/39), 1MO (22/39), and 1YR (17/39). Attrition was not significantly related to demographic variables (age, sex, concussion history) or clinical variables (symptom severity, time to RTP) examined in this study, based on Spearman correlations with adjustment at a false discovery rate (FDR) of 0.05 across imaging sessions.

As a control group, 71 athletes were also consecutively recruited and imaged at the start of their competitive season. All athletes in the study completed baseline assessments with the Sport Concussion Assessment Tool (SCAT) (15, 16) before the beginning of their athletic seasons. Athletes diagnosed with a concussion also completed SCAT assessments at acute injury and at the time of RTP. None of the athletes in this study had a history of neurological or psychiatric diseases or sensory/motor impairments, and none of the concussed athletes experienced loss of consciousness or posttraumatic amnesia. This study was carried out in accordance with the recommendations of the Canadian Tri-Council Policy Statement 2 (TCPS2) and with approval of the research ethics boards at the University of Toronto and St. Michael's Hospital, with written informed consent given by all participants in accordance with the Declaration of Helsinki.

### Magnetic Resonance Imaging

Athletes were imaged at St. Michael's Hospital using a research-dedicated MRI system operating at 3 T (Magnetom Skyra, Siemens, Erlangen, Germany) with the standard multichannel head receiver coil. Structural imaging included the following protocol: three-dimensional T1-weighted magnetization prepared rapid acquisition gradient echo imaging [MPRAGE: inversion time (TI)/echo time (TE)/repetition time (TR) = 1,090/3.55/2,300 ms, flip angle (FA) = 8°, 192 sagittal slices with field of view (FOV) = 240 × 240 mm, 256 × 256 pixel matrix, 0.9 mm slice thickness, 0.9 × 0.9 mm in-plane resolution, with bandwidth (BW) = 200 Hz/px], fluid attenuated inversion recovery imaging (FLAIR: TI/TE/TR = 1,800/387/5,000 ms, 160 sagittal slices with FOV = 230 × 230 mm, 512 × 512 matrix, 0.9 mm slice thickness, 0.4 × 0.4 mm in-plane resolution, BW = 751 Hz/px) and susceptibility-weighted imaging (SWI: TE/TR = 20/28 ms, FA = 15°, 112 axial slices with FOV = 193 × 220 mm, 336 × 384 matrix, 1.2 mm slice thickness, 0.6 × 0.6 mm in-plane resolution, BW = 120 Hz/px). To rule out potential structural abnormalities, the MPRAGE, FLAIR, and SWI scans were reviewed in a two-step procedure, with initial inspection by a certified MRI technologist during the imaging session and later review by a neuroradiologist with clinical reporting if abnormalities were identified. No abnormalities (white matter hyperintensities, contusions, microhemorrhage, or statistical outliers) were found for the concussed athletes and controls in this study.

For CVR measurements, BOLD fMRI data were acquired during a respiratory challenge task, via multi-slice T2^*^-weighted echo planar imaging (EPI: TE/TR =30/2000 ms, FA = 70^o^, 32 oblique-axial slices with FOV = 200 × 200 mm, 64 × 64 matrix, 4.0 mm slice thickness with 0.5 mm gap, 3.125 × 3.125 mm in-plane resolution, BW = 2,298 Hz/px). The BOLD fMRI data were processed and analyzed using a combination of Analysis of Functional Neuroimages (AFNI; https://afni.nimh.nih.gov) and in-house software. After discarding the first four volumes to allow scans to reach equilibrium, preprocessing included rigid-body motion correction (AFNI *3dvolreg*), removal of outlier scan volumes using the SPIKECOR algorithm (nitrc.org/projects/spikecor), slice-timing correction (AFNI *3dTshift*), spatial smoothing with a 6 mm full width at half maximum (FWHM) isotropic 3D Gaussian kernel (AFNI *3dmerge*) and regression of motion parameters and linear-quadratic trends as nuisance covariates. For motion parameter regression, principal component analysis was performed on the six rigid-body movement parameters, and the first two components were used as nuisance regressors (consistently accounting for >85% of variance). Finally, a low-pass Butterworth filter was applied to each voxel time series, with passband edge at 0.08 Hz and stopband edge at 0.10 Hz. This provides a conservative method for filtering physiological fluctuations outside of the neurovascular range of response. To perform group-level analyses, the fMRI data were then co-registered to a common anatomical template using the FMRIB Software Library (FSL) package (https://fsl.fmrib.ox.ac.uk). The FSL *flirt* algorithm was used to compute the rigid-body transform of the mean functional volume for each athlete to their T1-weighted anatomical image, along with the 12-parameter affine transformation of the T1 image for each athlete to the MNI152 template. The transformation matrices were then concatenated and the net transform applied to the functional imaging data, which was resampled at 3 mm × 3mm × 3 mm resolution.

To restrict analyses to gray matter, subject T1-weighted anatomical images were segmented and co-registered to the MNI152 template space using the *fslvbm* protocol (fsl.fmrib.ox.ac.uk/fsl/fslwiki/FSLVBM): (1) *bet* was used to create a mask of brain voxels and *fast* was used to create partial volume segmentations of gray matter (GM), white matter (WM), and cerebrospinal fluid (CSF); (2) the subject GM maps were co-registered affinely to FSL's 152T1 probabilistic GM template using *flirt* and a symmetric, study-specific template obtained by averaging transformed GM maps, which was then reaveraged with flipped left/right orientations; (3) the subject GM maps were then co-registered non-linearly to the mean affine template using *fnirt* and the symmetric, study-specific template recalculated; (4) this step was repeated once more using *fnirt* and the mean non-linear template was updated. The net transforms were also applied to WM and CSF maps to obtain similar mean templates, all of which were resampled into 3 mm × 3 mm × 3 mm resolution. A 6 mm FWHM Gaussian smoothing kernel was applied to the mean tissue templates, followed by voxel-wise renormalization to ensure that the tissue probabilities sum to unity. We then obtained a mask of regions with *p*(GM) > 0.33 (i.e., greater than chance probability of being GM in a three-compartment model), to restrict CVR analyses to gray matter voxels.

During the fMRI scans, respiratory responses were recorded using the cushion available with the MRI system. The fMRI task paradigm was adapted from previous robust methodology (12, 17). Participants received task instructions visually projected on a display screen, viewed through an angled mirror mounted on the head coil. Participants performed three task phases: controlled breathing (CB), breath holding (BH), and normal breathing (NB). The CB phase consisted of six repetitions of a 6-s full-breathing cycle, for a total duration of 36 s. Participants were guided visually by a circle that changed size, increasing linearly during inspiration (3 s) and decreasing linearly during expiration (3 s). This was followed by the BH phase, with participants instructed to hold their breath for 16 s with a visual countdown displayed. Finally, participants were instructed to perform the NB phase by breathing normally for a duration of 8 s with a visual countdown displayed. This CB–BH–NB “epoch” of 60 s duration was repeated six times for a CVR recording session of 6 min. Each BH task started at the end of a CB expiration cycle, and participants were instructed not to inhale prior to the BH task, to reduce this potential source of variation in BOLD response (18).

Our previously implemented protocol (12) was used to identify and remove outlier scans from sessions with potential participant compliance issues. First, the respiratory trace was inspected: a respiratory “template” time series was calculated by averaging across all controls, using a robust M-estimator (tuning parameter *k* = 1.35) (19). Any participant with a respiratory trace having Pearson correlation with the template of ρ < 0.30 was discarded as an outlier. For the remaining participants, the analogous process was repeated for the mean BOLD time series (averaged over all gray matter voxels). Using this protocol, three sessions were excluded: one control scan had an outlier respiratory response (ρ = 0.085), and one control and one concussed athlete scan had outlier BOLD responses (ρ = 0.123 and −0.067, respectively).

### Participant Demographics

The demographics for control and concussed athlete groups were reported, including age, sex, and prior concussion history, along with time to RTP for concussed athletes [i.e., the number of days from concussion event to symptom resolution following a graded exertional protocol (1)]. In addition, SCAT symptom data were reported at preseason baseline for both groups, as well as acute injury and RTP for concussed athletes. The SCAT data included a symptom severity score obtained by summing across the 22-item symptom scale, with each item receiving a 7-point Likert scale rating. A total symptoms score was also obtained by counting the number of symptoms with non-zero ratings. For concussed athletes, symptoms at acute injury and RTP were compared to controls and their own preseason baseline values using two-sample and paired-measures Wilcoxon tests, respectively. Significant tests were reported after adjusting for multiple comparisons at an FDR of 0.05. Unless otherwise noted, group statistics are summarized by the median with upper and lower quartiles (Q1, Q3).

Task compliance was also evaluated and compared between the control and concussed groups, based on the acquired respiratory trace data. The intraparticipant reliability was computed as the average Pearson correlation between respiratory traces from the 6*x*(60 s) task epochs, averaged over the 15 unique pairwise correlations. The interparticipant reliability was also computed as the correlation of the full respiratory trace for each athlete with the respiratory “template” time series defined in *Magnetic Resonance Imaging* above. The distribution of correlation values was compared between control and concussed groups using two-sample Wilcoxon tests.

### Normal Respiratory Response

The normal BOLD response during the respiratory challenge paradigm was first described for the control group. In our previous study (12), the BOLD response to respiratory challenge was found to exhibit a high degree of spatial uniformity; therefore, the analyses in this paper focus on average whole-brain BOLD response. For each participant, the six 60-s task epochs were averaged, producing a mean spatiotemporal BOLD matrix **X** (voxel × time) of the task sequence CB–BH–NB. For each participant, the mean BOLD temporal response ${\bar{\text{x}}}_{t}$ (time × 1) was then calculated from **X**, by averaging over all gray matter voxels for each task time point. The control group average of ${\bar{\text{x}}}_{t}$ was plotted, along with 95% confidence intervals (95% CIs) of the mean, obtained via bootstrap resampling (1,000 iterations). In addition, to show the spatial distribution of respiratory task-related activation, the mean BOLD spatial response ${\bar{\text{x}}}_{s}$ (voxels × 1) was calculated from **X**, by averaging over all time points for each voxel. The brain map of the control group average of ${\bar{\text{x}}}_{s}$ was then plotted, along with the standard error of the mean.

Subsequent analyses tested for effects of demographic variables on the BOLD response of the control group. A set of general linear models (GLMs) were fit, regressing each task time point in ${\bar{\text{x}}}_{t}$ against demographic covariates of age (integer), sex (binary), and history of concussion (binary). Bootstrapping was used to generate empirical distributions on the regression coefficients *b* and calculate *p*-values on the coefficient values. The effects of demographic covariates were then evaluated, with significance testing conducted at an FDR of 0.05. As demographic effects were non-significant for controls, the standardized effect sizes were reported as bootstrap ratios (BSRs; a normally distributed statistic, defined as the bootstrap mean/standard error), and were summarized in terms of the mean and range across task time points.

### Longitudinal Effects of Concussion

To model longitudinal change in the BOLD response of concussed athletes in the presence of missing data (see *Study Participants*), the effects of imaging session on BOLD response was estimated for each task time point of ${\bar{\text{x}}}_{t}$ within a linear mixed effects model (LMM), which modeled fixed effects of imaging sessions (SUB, RTP, 1MO, 1YR) relative to ACU and subject-specific random-effects intercepts. The models were fitted using the Matlab R2017b *fitlme* package (The MathWorks, Natick, MA) with full covariance estimation using Cholesky parameterization. Analysis was done in a bootstrap resampling framework, where resampling units consisted of all scans for a given participant (1,000 iterations). This was used to obtain empirical *p*-values on the fixed-effect coefficients *b*. The task time points of ${\bar{\text{x}}}_{t}$ showing a significant effect of imaging session were then identified, at an FDR of 0.05. Next, the BOLD values were averaged across significant task time points averaged and reanalyzed with a bootstrapped LMM to obtain summary statistics, including fixed-effect coefficient *b* with a 95% confidence interval (95% CI), BSR, and *p*-value for each imaging session. In addition, a spatial map of the brain regions showing longitudinal effects was obtained by repeating the above analysis at each voxel, restricted to the imaging session contrast of greatest effect. A voxel-wise threshold of *p* = 0.005 was applied, followed by cluster-size thresholding at *p* = 0.05, using AFNI *3dFWHMx* to estimate spatial smoothness of the fMRI maps, which was used as input to AFNI *3dClustSim* to determine the minimum cluster size threshold.

For the averaged BOLD values, obtained from task time points of ${\bar{\text{x}}}_{t}$ showing significant effects of imaging session, concussed athlete values were also compared to athletic controls. For each imaging session (ACU, SUB, RTP, 1MO, 1YR), concussed and control group means were compared. As the analyses of controls found no significant effects of age, sex, or concussion history (see *Normal Respiratory Response* and *Longitudinal Effects of Concussion*), unadjusted group means of the concussed and control cohorts were compared using two-sample bootstrap tests (1,000 iterations). For all analyses, the mean effect and 95% CIs were reported, along with BSRs and *p*-values, with significant task time points identified at an FDR of 0.05. To mitigate potential bias and loss of efficiency due to missing data, bootstrap analysis was combined with multiple imputation using the “Boot MI” approach of Schomaker and Heumann (20): bootstrap samples were drawn from the full dataset (including missing data), and for each sample, imputation was performed *M* = 10 times to obtain 10 coefficient estimates, which were averaged to produce a single point estimate. The set of coefficient point estimates were then treated as a conventional bootstrap empirical distribution, from which summary statistics were calculated. Imputation was done using the fitted LMM to generate simulated mean BOLD values (see Appendix 2 for a comparison of MI and unimputed analysis results). In addition, a spatial map of the brain regions showing cross-sectional effects was obtained by repeating the above analysis at each voxel, restricted to imaging session of greatest effect. Significant brain regions were then identified using the cluster-based thresholding procedure described previously.

### Effects of Clinical Covariates

Secondary analyses of the concussed cohort tested whether longitudinal changes in the BOLD response were affected by clinical covariates. This included (1) total symptom severity at acute injury and (2) days to RTP. To account for high correlation between symptoms and recovery time (see *Demographic and Clinical Data* below), two orthogonal composite scores (CS) were defined. After the two variables were renormalized via inverse empirical distribution function and mean centered, composite score 1 (CS1) was the average of the variables (total symptom severity + days to RTP), quantifying overall severity of clinical outcome. Composite score 2 (CS2) was the difference (total symptom severity – days to RTP), quantifying discrepancy between the two measures of concussion outcome (i.e., CS2 > 0 denotes high symptom burden but rapid recovery, and CS2 < 0 denotes the converse).

The effects of these covariate on BOLD values were assessed within the previously established bootstrapped LMM framework. In addition to the fixed-effect covariates of imaging session (SUB, RTS, 1MO, 1YR), the model was augmented by adding covariates for interaction effects of CS1 and CS2 with each imaging session. We then computed the contrast of interaction covariates for imaging sessions (SUB, RTP, 1MO, 1YR) relative to ACU. These interaction contrasts quantified the effects of clinical covariates on BOLD longitudinal changes between imaging sessions. Bootstrap resampling was then done to obtain empirical *p*-values on the fixed-effect coefficient contrasts *b*′. The task time points of ${\bar{\text{x}}}_{t}$ showing a significant interaction contrast were then identified, at an FDR of 0.05. Next, the BOLD values were averaged across significant task time points and reanalyzed with a bootstrapped LMM to obtain summary statistics, including fixed-effect coefficient contrast *b*′ with a 95% CI, BSR, and *p*-value for each imaging session. In addition, a spatial map of the brain regions showing CS interaction effects was obtained by repeating the above analysis at each voxel, restricted to the imaging session contrast of greatest effect. Significant brain regions were identified using the cluster-based thresholding procedure described previously.

## Results

### Participant Demographics

Table 1 reports demographic and clinical data for the control and concussed groups, which consisted of balanced samples of male and female athletes and individuals with and without prior history of concussion. Control athletes with a history of concussion reported a median of 1 prior concussion (1, 2), with the most recent occurring a median of 29 months (12, 48) before imaging. Concussed athletes with a history of concussion reported a median of 2 prior concussions (1, 3), with the most recent occurring a median of 38 months (24, 67) before imaging, but neither variable differed significantly from controls (*p* = 0.240, *p* = 0.417, respectively). For concussed athletes at acute injury, total symptoms and symptom severity were significantly elevated relative to the controls and their own baseline (*p* ≤ 0.001, for all tests). In contrast, at RTP, both symptom measures were no longer significantly elevated (*p* ≥ 0.981, for all tests). Among concussed athletes, the acute severity scores showed substantial variability across participants, as did time to RTP, with the variables having moderately high Spearman correlations (ρ = 0.690; 95% CI = 0.528–0.815; *p* < 0.001). None of the concussed athletes in this study had acquired a new concussion between ACU and 1YR imaging sessions, and all athletes had returned to normal school and/or work, social, and sport activities following RTP.

**Table 1:** Demographic data for athletes with concussion and controls, along with symptom scores, based on the sport concussion assessment tool (SCAT).

	**Control**	**Concussion**		
Age (mean ± SD)	20.2 ± 2.1		20.8 ± 2.2	
Female	37/71 (52%)		18/39 (46%)	
Previous concussions	32/71 (45%)		20/39 (51%)	
Days to RTP	–		31 (18, 64)	
		**Baseline**	**Acute**	**RTP**
Total Symptoms	2 (0, 4)	3 (2, 4)	10 (5, 16)^**^	1 (0, 2)
Symptom Severity	3 (0, 6)	3 (2, 10)	19 (6, 37)^**^	1 (0, 3)

The SCAT scores are represented as the median (Q1, Q3).

** *Significant difference in scores for the acute time point, relative to all other groups*.

Evaluating task compliance based on respiratory trace data, the within-participant reliability was relatively high for both controls [median: 0.711 (0.624, 0.770)] and concussed athletes at acute injury [0.708 (0.600, 0.777)], with similar values at subsequent imaging sessions. Similarly, the interparticipant reliability was relatively high for both controls [median: 0.749 (0.653, 0.831)] and concussed athletes at acute injury [median: 0.740 (0.640, 0.819)], with comparable values at later imaging sessions. Comparing the distributions of correlations, there were no significant reductions in task compliance for concussed athletes relative to controls (*p* ≥ 0.353, for both compliance measures and all imaging sessions).

### Normal Respiratory Response

**Figure 1:**
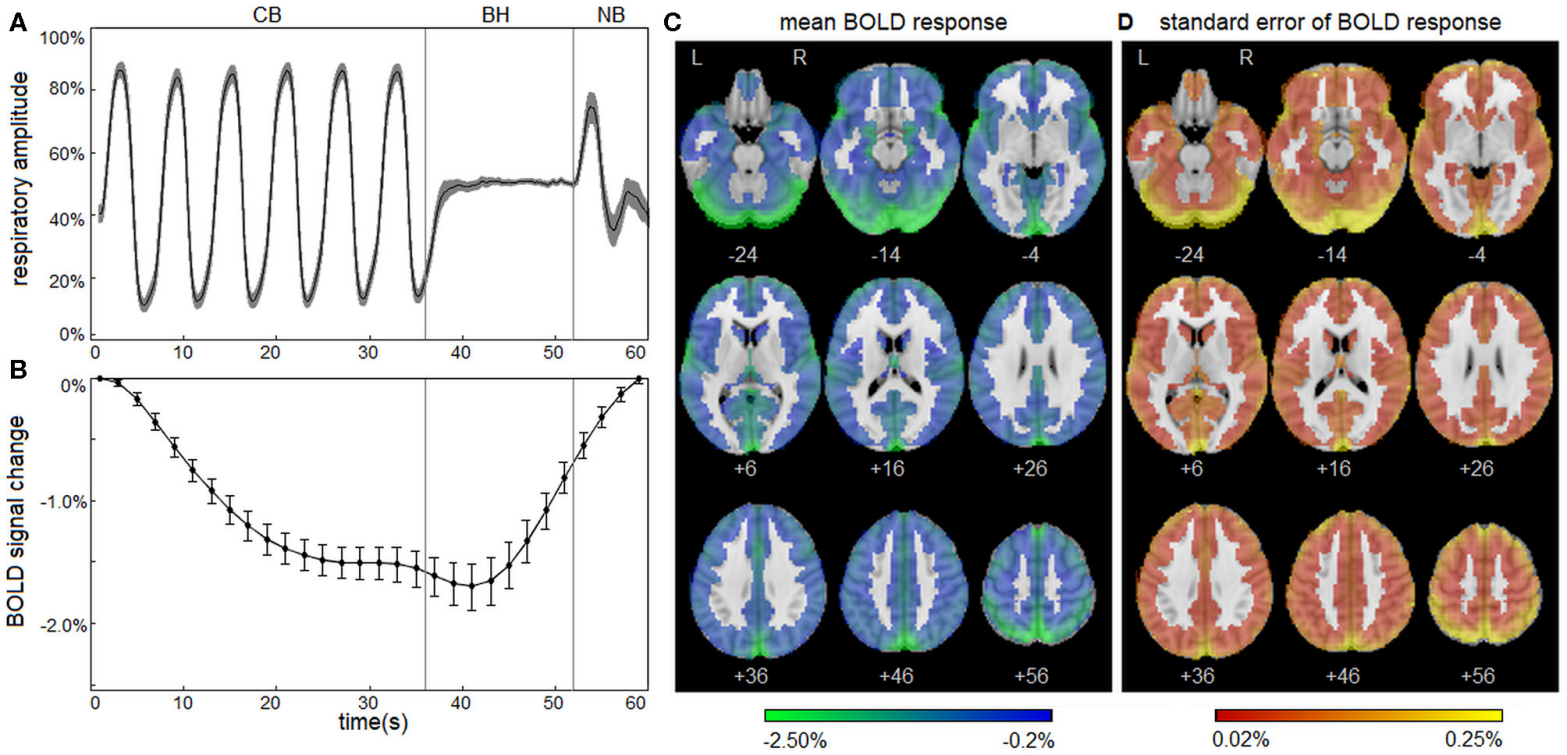
Blood-oxygenation-level-dependent (BOLD) response to the respiratory task for control athletes. **(A)** The group average of the mean respiratory trace, normalized to the range of 0–1 and downsampled to intervals of 0.20 s. **(B)** The group average of the mean BOLD temporal response ${\bar{\text{x}}}_{t}$. Shaded areas and error bars denote the bootstrapped 95% confidence intervals of the mean. **(C)** The group average of the mean BOLD spatial response ${\bar{\text{x}}}_{s}$. **(D)** The standard error of the group average ${\bar{\text{x}}}_{s}$. Plots depict a highly consistent respiratory trace and BOLD response to the task for athletic controls.

Figure 1 depicts the normal response to the respiratory task for control athletes, including group averages for the respiratory trace (Figure 1A) and the mean BOLD temporal response ${\bar{\text{x}}}_{t}$ (Figure 1B). The controls show highly consistent respiratory traces and BOLD temporal response curves with narrow 95% CIs, which is consistent with the task compliance reported in *Participant Demographics* above. During the initial part of the CB task phase, mean BOLD signal decreased from *t* = 1–27 s. This was followed by a negative plateau of 1.5% from *t* = 29–41 s for the remainder of CB and a slight decrease to 1.7% at the start of BH. Subsequently, during BH and throughout NB, mean BOLD signal increased from *t* = 43–59 s, returning to reference levels by the end of the task. The mean BOLD temporal response ${\bar{\text{x}}}_{t}$ had strong Pearson correlations with voxelwise time series throughout the brain; the distribution of voxelwise correlations, averaged over participants, was uniformly high [median: 0.898 (0.822, 0.932)], indicating spatial uniformity in the shape of the temporal BOLD response. The group average is also depicted for the mean BOLD spatial response ${\bar{\text{x}}}_{s}$ (Figure 1C), showing that the amplitudes of response are somewhat heterogeneous across brain voxels [median: −0.87% (−1.16%, −0.67%)] with greatest response seen in occipital and parietal regions. In addition, the standard error of the mean for ${\bar{\text{x}}}_{s}$ is displayed (Figure 1D), with higher values seen in areas of high mean BOLD response, but generally an order of magnitude lower than the mean response [median: 0.08% (0.05%, 0.12%)]. These results confirm that the normal BOLD response to the respiratory task was highly consistent for control athletes. For the control group, the BOLD response showed no significant effects of age (mean absolute BSR of 0.39; range, 0.02–0.84), sex (mean absolute BSR of 0.79; range, 0.10–1.50) or history of concussion (mean absolute BSR of 1.14; range, 0.11–2.09) after adjusting for multiple comparisons at an FDR of 0.05.

### Longitudinal Effects of Concussion

**Figure 2:**
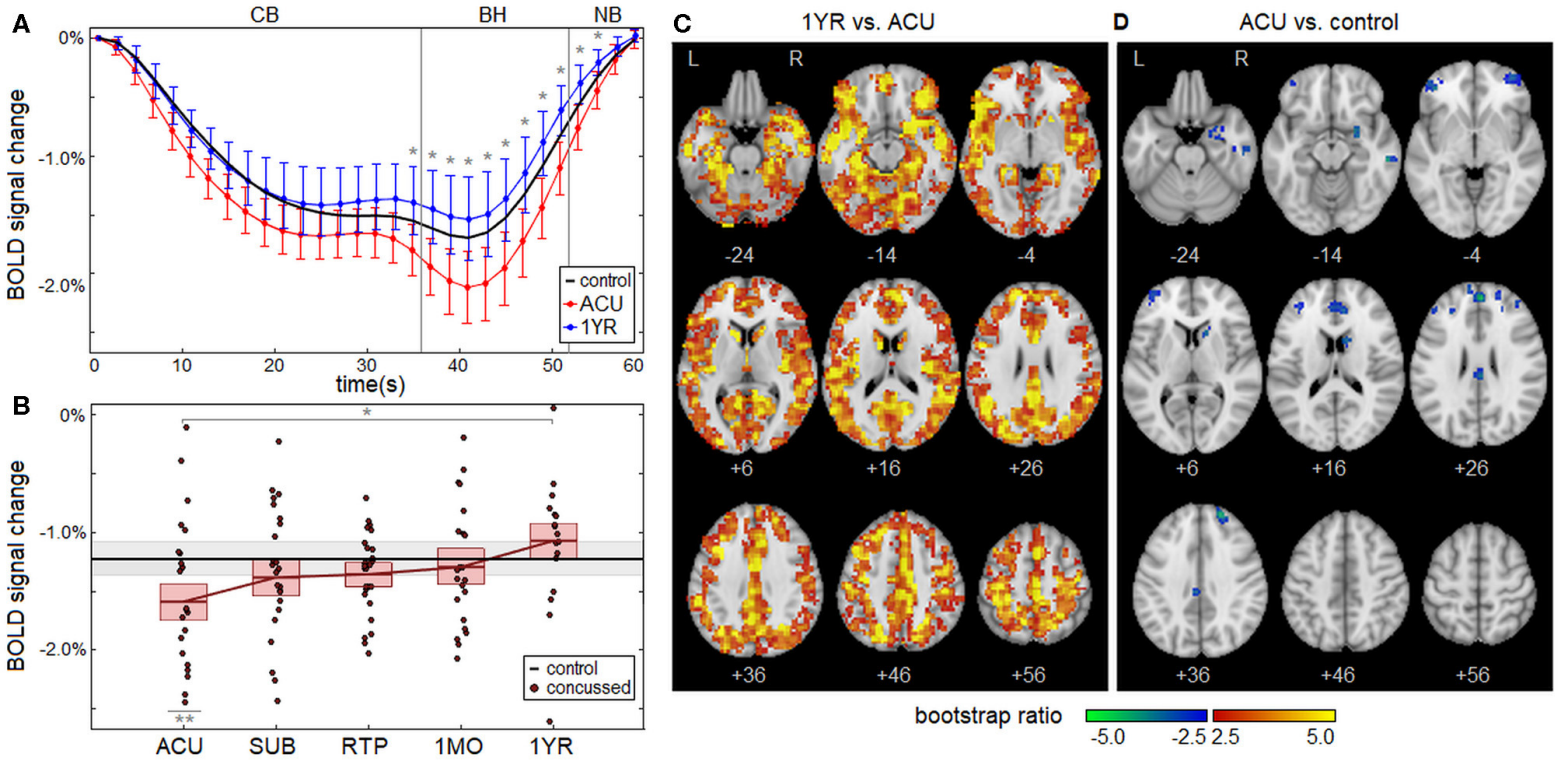
**(A)** The group average of the mean BOLD temporal response ${\bar{\text{x}}}_{t}$, plotted for concussed athletes at acute injury (ACU; red) and 1 year post-RTP (1 YR; blue), along with controls (black). Errorbars denote 95% confidence intervals (95% CIs) of the mean and “*” denotes task time points with a significant effect of imaging session, at an FDR of 0.05. **(B)** Subject BOLD values, averaged over significant task time points (35–55 s), plotted for each imaging session. The horizontal red lines denote group means, and boxes indicate bootstrapped 95% CIs of the means. The mean value for controls is plotted as the thick horizontal black line, with the corresponding 95% CI shaded in gray. In the plots, “*” denotes significant longitudinal change relative to ACU and “**” denotes significant cross-sectional difference relative to controls. **(C)** Brain regions showing significant differences in BOLD response of concussed athletes at 1 YR relative to ACU. **(D)** Brain regions showing significant differences in BOLD response of concussed athletes at ACU relative to control athletes.

Concussed athletes had an enhanced negative BOLD response to the respiratory task at ACU, with a gradual attenuation of effect over subsequent imaging sessions. The mean BOLD temporal response is plotted in Figure 2A for concussed athletes at ACU and 1YR, and for control athletes. At ACU, a greater negative response was seen relative to controls, whereas at 1YR, the response was slightly less negative than the controls. Significant longitudinal changes in BOLD response were seen at 1MO, for task time points *t* = 49–53 s, with more extensive effects at 1YR, for *t* = 35–55 s, which corresponds to the end of the CB task phase, all of BH and the early part of NB. Table 2 reports statistics of longitudinal effect, for BOLD responses averaged over significant task time points *t* = 35–55 s, which had uniformly positive coefficient values. The averaged BOLD values are also plotted in Figure 2B for each imaging session, showing the progressive reduction in BOLD response over time; it is only at 1YR that the mean response becomes less negative than the mean control value. Figure 2C shows significant longitudinal changes from ACU to 1YR at the voxel level, with increases throughout the brain. Cross-sectional bootstrap analysis (Table 2) determined that only the ACU imaging session was significantly different in BOLD response relative to controls. Figure 2D shows significant cross-sectional differences at ACU relative to controls at the voxel level, with spatially limited effects seen mainly in orbitofrontal, anterior cingulate, and middle frontal regions.

**Table 2:** Longitudinal effects of concussion on blood-oxygenation-level-dependent (BOLD) response, averaged over significant task time points (35–55 s).

	**Longitudinal (LMM)**				**Cross-sectional (2-sample)**			
	***b***	**95% CI**	**BSR**	***p***	**mean**	**95% CI**	**BSR**	***p***
ACU	–	–	–	–	−0.325	−0.551, −0.122	−3.41	<0.001^**^
SUB	0.207	−0.093, 0.488	1.34	0.164	−0.138	−0.367, 0.070	−0.98	0.208
RTP	0.239	−0.008, 0.496	1.85	0.060	−0.138	−0.323, 0.034	−0.88	0.128
1MO	0.294	0.042, 0.517	2.34	0.026	−0.078	−0.279, 0.113	−0.20	0.454
1YR	0.539	0.247, 0.873	3.52	<0.001^*^	0.143	−0.064, 0.355	1.64	0.524

(Left) Statistics from longitudinal linear mixed model (LMM) analyses, including fixed-effect coefficients b, 95% confidence intervals (95% CIs), bootstrap ratios (BSRs), and p-values. (right) Statistics from bootstrapped two-sample cross-sectional analyses, including mean, 95% CI, BSRs, and p-values. Longitudinal tests compare imaging sessions (SUB, RTP, 1MO, 1YR) to ACU, whereas cross-sectional tests compare imaging sessions (ACU, SUB, RTP, 1MO, 1YR) to athletic controls.

* Significant longitudinal change relative to ACU.

** *Significant cross-sectional difference relative to controls*.

### Effects of Clinical Covariates

**Figure 3:**
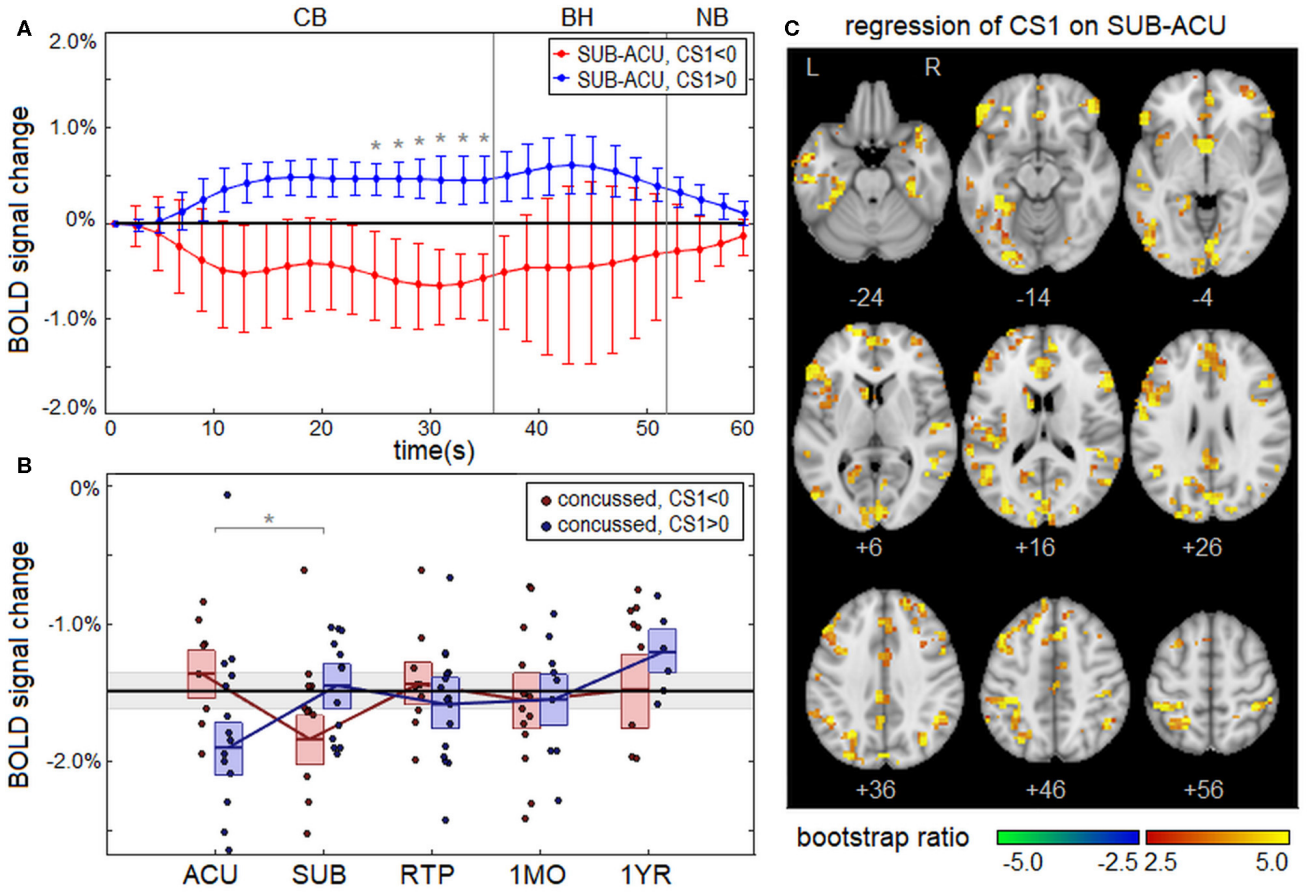
**(A)** Change in the group average of the mean BOLD temporal response ${\bar{\text{x}}}_{t}$ from ACU to SUB, plotted for athletes with low severity on clinical composite score 1 (CS1 < 0; red) and high severity (CS1 > 0; blue), along with the line of no effect (black). Errorbars denote 95% confidence intervals (95% CIs) of the mean and “*” denotes task time points with significant effect of CS1 on longitudinal BOLD change, at an false discovery rate (FDR) of 0.05. **(B)** Subject BOLD values, averaged over significant task time points (25–35 s), were plotted for each imaging session. The horizontal red/blue lines denote subgroup means, and boxes indicate bootstrapped 95% CIs of the means. The mean value for controls is plotted as the thick horizontal black line, with the corresponding 95% CI shaded in gray. In the plots, “*” denotes significant longitudinal change relative to ACU. **(C)** Brain regions showing significant effects of CS1 on the BOLD change from ACU to SUB.

Concussed athletes showed significant effects of clinical composite score CS1 (total symptom severity + days to RTP) on the longitudinal change in BOLD response from ACU to SUB; in contrast, no significant effects were identified for composite score CS2 (total symptom severity – days to RTP). The change in the mean BOLD temporal response (SUB – ACU) is plotted in Figure 3A separately for concussed athletes with low severity (CS1 < 0) and with high severity (CS1 > 0). The CS1 < 0 subgroup showed a negative BOLD signal change between sessions, whereas the CS1 > 0 subgroup showed a positive signal change. Significant effects of CS1 on the longitudinal change in BOLD response were seen for task time points *t* = 25–35 s, which corresponds to the late part of the CB task phase. Averaging over significant task time points from *t* = 25–35 s, which had uniformly positive coefficient values, a coefficient value of *b* = 0.238 was obtained (95% CI: 0.071, 0.361; BSR = 3.20; *p* < 0.001). The averaged BOLD values are plotted separately for CS1 < 0 and CS1 > 0 subgroups in Figure 3B. For the CS1 < 0 subgroup, the mean BOLD response was close to that of control athletes at ACU but became more negative at SUB, before returning to near normal values at later imaging sessions. For the CS1 > 0 subgroup, the mean BOLD response was most negative at ACU but increased to near-normal values for all imaging sessions afterwards. Figure 3C shows significant effects of CS1 on the longitudinal change from ACU to SUB at the voxel level. The effects were distributed throughout the brain, with large clusters particularly in occipital, inferior frontal, and anterior cingulate regions.

## Discussion

Concussion is associated with significant disturbances in brain physiology in the days following injury, including impairments in CVR. However, there is a paucity of research data concerning the long-term recovery of CVR, particularly with respect to medical clearance to RTP. This paper is the first to use BOLD fMRI to investigate CVR longitudinally from acute injury to 1 year post-RTP. The principal study findings were that (1) CVR was elevated at acute injury, based on an enhanced negative BOLD response to a respiratory task, with significant reductions in BOLD response at 1 year post-RTP; (2) significant differences in CVR relative to controls were detected only at acute injury; and (3) clinical covariates showed significant effects on the longitudinal change in CVR from acute to subacute injury.

The BOLD response was first examined in a control group of athletes without recent concussion. The group was demographically mixed (male and female, with and without history of concussion, drawn from different sports). Nevertheless, the BOLD response during the respiratory challenge task was highly consistent across participants. The BOLD response was similar to previous studies (12, 17, 21), with progressively more negative signal during the CB phase, followed by a plateau and subsequent return to baseline during BH and NB phases. The reduction in BOLD signal during CB is consistent with a hypocapnic state during cued deep breathing (17), while the increase in BOLD signal during BH is consistent with a hypercapnic state (21). Regression analyses found no significant effects of age, sex, or concussion history on BOLD response for the control group. These findings differ from existing literature, which had reported variations in CVR associated with healthy aging (22, 23) and sex (24). However, these studies were conducted in older non-athlete populations across a wider age span than the present study. The results of this study suggest that, for young athlete cohorts, CVR may be robust to demographic variations, and subgroups may be combined to improve the power to detect concussion effects, as was done in previous studies (9). However, for BOLD fMRI studies of CVR and concussion in non-athletes and wider age ranges, demographic confounds should still be carefully examined before evaluating the effects of concussion.

Turning now to concussed athletes, longitudinal analyses showed that the BOLD response to respiratory challenge did not significantly change between acute injury and time of RTP. This is aligned with previous BOLD fMRI literature, which found evidence of incomplete functional brain recovery at RTP, both for resting-state functional connectivity (25) and for task-related activation (26). These findings provide further evidence that biological recovery lags behind clinical symptom resolution. Limited changes were identified at 1 month post-RTP, whereas effects were most extensive at 1 year post-RTP, suggesting that CVR recovery is largely completed between these imaging sessions. The observed effects are consistent with a previous case study of mild TBI (11), which reported CVR abnormalities at 2 months post-injury that had resolved by 1 year post-injury. However, concussion effects identified in the present study were relatively small beyond the acute phase of injury, as only the first imaging session showed a significantly different CVR response compared to control athletes. These findings highlight the subtle but persistent nature of concussion-related alterations in CVR.

In this study, acutely concussed athletes had an enhanced negative BOLD response compared to controls, with greatest effects occurring at the end of the CB task phase, all of BH, and the early part of NB. The larger negative BOLD response seen at the end of CB suggests that the effects of concussion on acute CVR are greatest during sustained hypocapnic stimuli and involve enhanced vasoconstrictive activity. By contrast, despite the greater negative BOLD values at the initiation of BH, the BOLD signal had returned to baseline levels by the end of NB; thus, it is unlikely that vasodilatory capacity is impaired. In more severe TBI, vasoconstriction may serve to limit cerebral bleeds but increases the subsequent risk of ischemia (27). In the case of concussion, is it less clear, though, to what extent the observed effects represent a pathological and/or neuroprotective response to injury (28). Enhanced CVR during the early symptomatic phase of injury has been reported in our initial study of acute sport-related concussion (12) and in other concussion studies that used different CVR assessment protocols (3, 5, 10). In addition, there is evidence that the vasoconstrictive response is more preserved than the vasodilatory response after a concussion (5). However, concussion-related changes in BOLD response may be influenced by systemic factors other than vascular tone. Both blood pressure and cardiac output affect CBF (4), and the regulation of both may be impaired following a concussion (3, 29), leading to an altered BOLD response. Future research with more comprehensive peripheral monitoring of patients will be needed to disentangle these physiological factors. Interestingly, the BOLD response was slightly elevated relative to controls at 1 year post-RTP. Although the effect did not attain statistical significance, concussion may lead to subtle decrements in CVR over the long term. In this study, the effects of history of concussion were non-significant for controls, although this group had a median of 1 prior concussion. It is presently unclear whether multiple concussions have a cumulative long-term effect on CVR, making this an important area of further research.

The analysis of clinical covariates showed a significant effect on CVR recovery from acute to subacute injury. Individuals with greater acute symptom burden and prolonged recovery (CS1 > 0) had an enhanced negative BOLD response in the acute phase of injury; this is consistent with our previous findings, in which greater symptom severity was associated with enhanced BOLD response at acute injury (12). However, the CVR effects were attenuated in the subacute phase of injury, despite a lack of clinical resolution. This may be due to effects secondary to impaired blood flow regulation (e.g., excitotoxicity, edema) further contributing to delayed recovery in this group (30). In contrast, for individuals with low acute symptoms and rapid recovery (CS1 < 0), enhanced BOLD response was seen at the subacute phase of injury. This suggests that a delayed onset of CVR disturbances may be related to better clinical outcome after concussion. Further research should examine recovery of both symptoms and CVR in the subacute phase in greater detail, to better understand how these relationships evolve over time. The respiratory task time points significantly affected by CS1 occurred in the CB task phase. Therefore, enhanced vasoconstriction is associated with both the acuteness of injury and the severity of clinical outcome. The task time points associated with CS1 also occurred earlier in the CB phase (*t* = 25–35 s) than task time points showing main effects of time (*t* = 35–55 s). Intriguingly, this suggests that for individuals with more severe clinical outcome, acute CVR abnormalities appear earlier in the presentation of a sustained hypocapnic stimulus, reflecting a more reactive cerebrovascular system.

In terms of the spatial distribution of affected brain regions, significant longitudinal changes in CVR from acute injury to 1 year post-RTP were seen throughout the brain, exibiting a high degree of spatial uniformity. In contrast, significant cross-sectional differences between acutely concussed athletes and controls were far more spatially limited. This reduced sensitivity is likely driven by intersubject variability of baseline CVR values, which may be substantial and depends on the brain region of interest (9), and by the spatial heterogeneity of concussion effects, which has been previously seen in analyses of resting cerebral blood flow (31). Significantly affected brain regions were almost entirely frontal, which is likely due to the vulnerability of these regions to primary injury during concussive impacts (32, 33). Similarly, large frontal clusters showed significant effects of clinical score CS1 on CVR recovery, emphasizing that these brain regions may be particularly vulnerable to concussion-related changes in CVR, with consequent increases in both acute symptom severity and recovery time.

Although this is one of the most extensive studies of concussion and CVR to date, the findings should be interpreted in the context of the study limitations. The current respiratory challenge paradigm has the advantages of being easy to administer, inexpensive, and well-tolerated by even highly symptomatic athletes. However, it provides an indirect measure of CVR, which is typically quantified as a function of arterial carbon dioxide (CO_2_). Hence, there may be variability in our findings due to baseline variations in arterial CO_2_ levels (7). Baseline vascular tension may also affect the results (34), as a systematic difference in normocapnic vasodilatory status among concussed athletes may lead to over- or underestimation of the amplitude of CVR response during CB and BH phases of the task. Although the current paradigm did not control for variations in baseline oxygenation status, this was addressed in our previous study (12), which found CVR response to be unrelated to resting CBF among concussed athletes. As an additional limitation, breath holding was limited to 16 s intervals during the respiratory task, to ensure a good tradeoff between signal detection and task compliance (17). However, this limits our ability to detect signal changes that are associated with more prolonged intervals of hypercapnia. Future studies of long-term recovery may benefit from using blended gas delivery systems, to better control for physiological factors involved in CVR estimation and to assess the effects of concussion on more prolonged hypercapnic stimuli. Despite these limitations, the present study demonstrates the utility of a simple BOLD respiratory challenge paradigm for monitoring athletes post-concussion, which may be readily integrated into MRI studies to complement more commonly acquired resting-state data. Lastly, for the present study, CVR disturbances were identified by comparing concussed athletes to a demographically matched group of athletic controls. However, as previously noted, concussion effects may be underestimated due to intersubject variations in baseline CVR. In the future, it will be important to conduct prospective imaging studies that evaluate within-subject CVR changes from pre- to post-injury to control for this source of variability.

This study presents novel findings about the long-term recovery of CVR following a concussion. In particular, it provides evidence that the effects of concussion on cerebrovasculature include enhanced CVR during controlled breathing, with subtle but persistent effects that last beyond medical clearance to RTP but show evidence of resolution by 1 year post-RTP. In addition, a greater CVR response during the subacute phase of injury, compared to the acute phase, is associated with better clinical outcomes, including lower symptom severity and shorter recovery times. Although an indirect measure of true CVR, these study findings highlight the utility of simple respiratory challenge paradigms as a form of cerebrovascular “stress test” to evaluate impairments in vessel function.

## Data Availability Statement

The raw data supporting the conclusions of this article will be made available by the authors, without undue reservation.

## Ethics Statement

The studies involving human participants were reviewed and approved by The Canadian Tri-Council Policy Statement 2 (TCPS2) and the Research Ethics Boards of the University of Toronto and St. Michael's Hospital. The patients/participants provided their written informed consent to participate in this study.

## Author Contributions

NC, MH, and TS conceptualized and planned the study. NC performed analyses and manuscript preparation. TS, MH, and SG revised for critical intellectual content and assisted with interpretation of findings. All authors contributed to the article and approved the submitted version.

## Conflict of Interest

The authors declare that the research was conducted in the absence of any commercial or financial relationships that could be construed as a potential conflict of interest.
