# Supplementary material for: Cerebrovascular Reactivity After Sport Concussion: From Acute Injury to 1 Year After Medical Clearance
Source: Front Neurol. 2020 Jul 14;11:558. doi: 10.3389/fneur.2020.00558 (PMC7371921; doi:10.3389/fneur.2020.00558)
Supplement: Supplementary file 1 [file Data_Sheet_1.DOCX]

**Appendix 1: athlete numbers by sport**

**Table S1:** athlete numbers by sport, for both male (M) and female (F) groups, for N=71 controls and N=39 concussed athletes.

| **CONTROL** | **CONCUSSION** |
| --- | --- |
| --  Lacrosse (7M / 2F)  Basketball (5F)  Rugby (3M / 2F)  Football (7M)  Soccer (3M / 4F)  Hockey (10M / 14F)  Volleyball (3M / 10F)  --  --  Squash (1M) | Water polo (1F)  Lacrosse (2M)  Basketball (2M)  Rugby (5M / 9F)  Football (4M)  Soccer (1F)  Hockey (6M / 5F)  Volleyball (2M)  Mountain biking (1F)  Rowing (1F)  -- |
