# Supplementary material for: Cerebrovascular Reactivity After Sport Concussion: From Acute Injury to 1 Year After Medical Clearance
Source: Front Neurol. 2020 Jul 14;11:558. doi: 10.3389/fneur.2020.00558 (PMC7371921; doi:10.3389/fneur.2020.00558)
Supplement: Supplementary file 2 [file Data_Sheet_2.docx]

**Appendix 2: Effects of Imputation**

To determine whether main study findings were significantly altered by methods of correcting for missing data, the results of longitudinal analyses (in which LMM implicitly account for missing data) and cross-section analyses (in which missing values are imputed using the fitted LMM) were compared to similar analyses performing pairwise deletion (PD; removing only subjects with missing values from time point being analyzed). We did not compare against listwise deletion (LD; removing subjects with missing data from any time point), as there was insufficient complete longitudinal data to permit full bootstrapped parameter estimation. Longitudinal effects were modelled at each post-acute time point (SUB, RTP, 1MO and 1YR) by performing simple General Linear Model (GLM) analyses of the BOLD change relative to ACU. Cross-sectional analyses were performed in the same manner as for imputed data. In both bases, missing values were excluded prior to analysis, based on the PD approach.

As shown in supplemental Table S2 below, the non-imputed approach shows similar coefficient signs and comparable magnitudes as the main study findings of Table 2, for both longitudinal and cross-sectional analyses. However, the PD approach tends to show wider error bounds and reduced effect sizes (e.g., smaller bootstrap ratios) compared to imputed methods, with modest reductions for longitudinal analysis and greater reductions for cross-sectional analysis. This indicates that the direction of effect is not heavily dependent on the method chosen to handle missing data, but there is a substantial decrease in sensitivity for non-imputed analysis.

**Table S2**: longitudinal effects of concussion on BOLD response, averaged over significant task time points (35 to 55 s), with pairwise deletion (PD) of missing data. (left) Statistics from longitudinal general linear model (GLM) analyses, including regression coefficients $b$, 95% confidence intervals (95% CIs), bootstrap ratios (BSRs) and p-values. (right) Statistics from bootstrapped 2-sample cross-sectional analyses, including mean, 95%CI, BSRs and p-values. Longitudinal tests compare imaging sessions (SUB, RTP, 1MO, 1YR) to ACU, whereas cross-sectional tests compare imaging sessions (ACU, SUB, RTP, 1MO, 1YR) to athletic controls.

|  | Longitudinal (GLM) | | | | Cross-sectional (GLM) | | | |
| --- | --- | --- | --- | --- | --- | --- | --- | --- |
|  | *b* | 95%CI | BSR | *p* | *b* | 95%CI | BSR | *p* |
| ACU | -- | -- | -- | -- | -0.321 | -0.548, -0.080 | -2.44 | 0.016 |
| SUB | 0.152 | -0.233, 0.506 | 0.82 | 0.408 | -0.080 | -0.368, 0.143 | -0.58 | 0.597 |
| RTS | 0.275 | -0.011, 0.572 | 1.81 | 0.064 | -0.085 | -0.306, 0.103 | -0.80 | 0.516 |
| 1MO | 0.268 | -0.027, 0.551 | 1.81 | 0.078 | -0.014 | -0.270, 0.274 | -0.11 | 0.839 |
| 1YR | 0.763 | 0.401, 1.165 | 3.93 | <0.001 | 0.179 | -0.140, 0.480 | 1.03 | 0.242 |
