# Supplementary material for: Cerebrovascular Reactivity After Sport Concussion: From Acute Injury to 1 Year After Medical Clearance
Source: Front Neurol. 2020 Jul 14;11:558. doi: 10.3389/fneur.2020.00558 (PMC7371921; doi:10.3389/fneur.2020.00558)
Supplement: Supplementary file 3 [file Data_Sheet_3.DOCX]

**Appendix 3: mean spatial BOLD response by task phase**

In this section, the mean BOLD spatial response is shown in greater detail, for both control and concussed athletes. For each brain voxel and participant, we calculated the mean % BOLD signal change during different phases of the respiratory task, including controlled breathing (CB; from t=0 to 36 s), breath hold (BH; from t=37 to 53 s) and normal breathing (NB; from t=54 to 60 s). The corresponding brain maps were then averaged across participants and representative brain slices plotted in Figure S1 below. Consistent with the results shown in Figure 2, concussed athletes tended to have greater negative BOLD values at ACU compared to controls, with effects attenuating over subsequent imaging sessions.


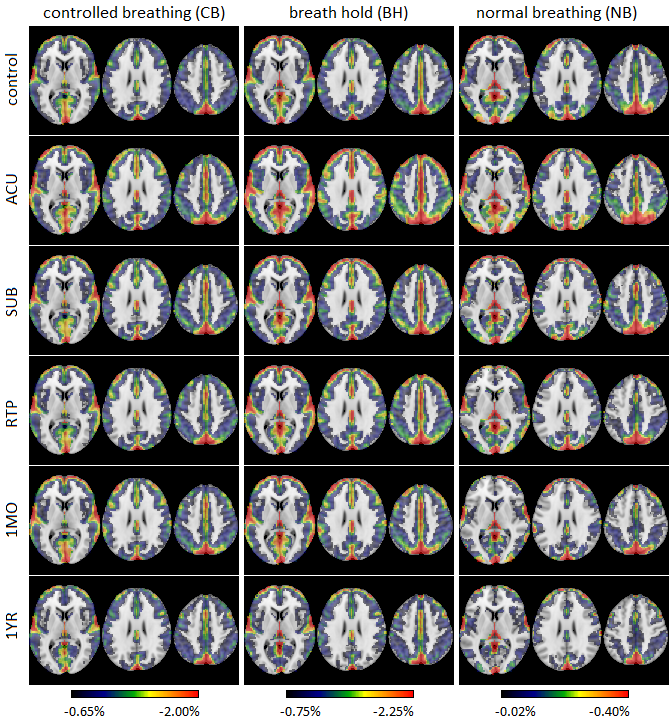


**Figure S1**: The group averages of the mean BOLD spatial response ${\bar{\boldsymbol{x}}}_{s}$, calculated separately for the respiratory task conditions of controlled breathing (CB), breath hold (BH) and normal breathing (NB). Mean plots are shown for controls and for concussed athletes, at acute injury (ACU), subacute injury (SUB), return to play (RTP), one month post-RTP (1MO) and one year post-RTP (1YR).
